# A New Methodology to Estimate Drug Cost Avoidance in Clinical Trials: Development and Application

**DOI:** 10.3389/fonc.2022.889575

**Published:** 2022-06-08

**Authors:** Sebastián García-Sánchez, Roberto Collado-Borrell, Eva González-Haba, José Luis Revuelta-Herrero, Vicente Escudero-Vilaplana, María Belén Marzal-Alfaro, María Norberta Sánchez-Fresneda, Ana Mur-Mur, Ana Herranz, Miguel Martín, María Sanjurjo

**Affiliations:** ^1^ Pharmacy Department, Hospital General Universitario Gregorio Marañón, Instituto de Investigación Sanitaria Gregorio Marañón (IiSGM), Madrid, Spain; ^2^ Medical Oncology Department, Hospital General Universitario Gregorio Marañón, IiSGM, Madrid, Spain

**Keywords:** clinical trial, antineoplastic agents, drug acquisition, drug cost avoidance, cost analysis

## Abstract

**Background:**

Oncology clinical trials can lead to relevant financial savings in drug acquisition for healthcare providers. Considerable methodological heterogeneity is observed among previous studies estimating these savings.

**Methods:**

We developed a methodology to estimate the economic benefit obtained from the enrollment of patients into clinical trials through the analysis of drug cost avoidance. We designed a decision algorithm to determine if a clinical trial is associated with drug cost avoidance. This algorithm is based on five scenarios according to the availability or not of standard treatment, the presence or absence of a control arm (placebo or active treatment), and the provider of the medication. We considered as reference the cost of the standard treatment that the patient would have received in routine clinical practice. We standardized drug doses and treatment durations according to the literature. Costs were considered from a National Health System perspective. We applied this methodology at a single, research-active University Hospital in 2019. A cost avoidance analysis per trial and patient was carried out on cancer patients.

**Results:**

We analyzed 140 trials in which 198 patients were recruited. Drug cost avoidance was found in 120 trials (85.7%). The estimated total drug cost avoidance amounted to over €3,200,000. Melanoma and genitourinary tumors were the tumor types associated with the highest cost avoidance. The average drug cost avoidance per patient was €16,245.

**Conclusion:**

We describe a standardized method to estimate drug cost avoidance in clinical trials. We have applied it to all ongoing oncology clinical trials in our center. This methodology could be valuable for other centers to analyze the potential saving of clinical trials.

## Introduction

Clinical trials are a key element in the development of new drugs, as they provide evidence about the efficacy and safety of new treatments before and even after their approval. Patients enrolled in clinical trials may benefit from the possibility of accessing therapies not yet available to the public or receiving treatment when no other therapeutic option is available. Clinical trials also benefit healthcare professionals, administrations, and society because they contribute to research and scientific knowledge development (1). From an institutional perspective, these benefits often contrast with the increasing demand for material and human resources that clinical trials require for their development, so there is a concern about their profitability from an economic point of view (2). Also, the innovative molecules derived from positive registration trials are generally associated with a cost increase for healthcare systems (3).

In the United States, cancer-attributable medical care costs were $183 billion in 2015 and they are projected to increase by over 30 percent to 2030, up to $246 billion (4). An essential part of this increase is explained by the significant rise in the cost of antineoplastic drugs. Likewise, in Europe, the expenditure on cancer medicines has tripled from €10 billion to €32 billion between 2005 and 2018 (5). In Spain, the consumption of antineoplastic drugs represented a total cost of €1,717 million in 2015, corresponding to about 36% of direct costs of cancer, 2.6% of public health expenditure, and 0.16% of the Spanish GDP (6). In addition, the number of active oncology clinical trials is also increasing. In Europe, the total of trials increased by 33% between 2010 and 2018. The increase was remarkable in early-phase trials (I-II; 61%) (7). Spain is the first European country by number of clinical trials carried out, of which oncology trials represent a growing percentage (8). Only during 2019, the Spanish Agency of Medicines and Health Products authorized 309 new clinical trials for patients with cancer.

Public health spending has steadily increased in recent years due to the improvements in the quality of healthcare systems, the use of innovative treatments, and the aging of the population. Sustainability has become a major issue for healthcare systems worldwide, and initiatives like the Quadruple Aim are an effort to optimize healthcare. This framework is focused in four overarching goals: improving the individual experience of care, improving the experience of providing care, improving the health of populations, and reducing the per capita cost of healthcare (9). Healthcare systems are implementing several strategies to improve efficiency of hospital drug spending, preventing unnecessary costs without diminishing or even improving the quality of care provided to patients (8).

Among the strategies of drug cost-containment, the potential savings that the enrollment of patients in clinical trials may offer to both patients and organizations should be remarked. In sponsored clinical trials, for example those carried out by the pharmaceutical industry, investigational drugs are provided by the sponsor and therefore, their cost is supported neither by the patient or the healthcare provider. Other interventions such as scans, laboratory tests, and other interventions can also be covered or reimbursed by the sponsor. Recent works have estimated the avoided cost associated with investigational drugs (10–17). Nevertheless, we believe there is a lack of a practical, reproducible, and structured methodology to estimate these savings and establish comparisons among institutions and organizations.

Therefore, the main objective of our work was to design a feasible and reproducible methodology to estimate the direct economic savings for a public healthcare institution obtained from the participation of oncology patients in clinical trials through the analysis of drug cost avoidance. Based on this methodology, the secondary objectives of this work were to estimate the economic impact for our institution of patient enrollment in oncology clinical trials and determine the tumor types in which this impact was most relevant.

## Methods

### Development of an Algorithm to Determine if a Clinical Trial Is Associated With Drug Cost Avoidance

Drug cost avoidance was defined as any expenditure that would have been made to procure drugs, but that was not made because of a specific trial-related intervention (18). Three variables were identified as key for determining if the participation of a patient in a clinical trial involves drug cost avoidance:

* Standard therapy: treatment that a patient would have received in our center in routine clinical practice if the patient had not been enrolled in the clinical trial.
* Investigational medicinal product: a medicinal product being tested or used as a reference, including a placebo, in a clinical trial (19).
* Provider of the medication.

**Figure 1:**
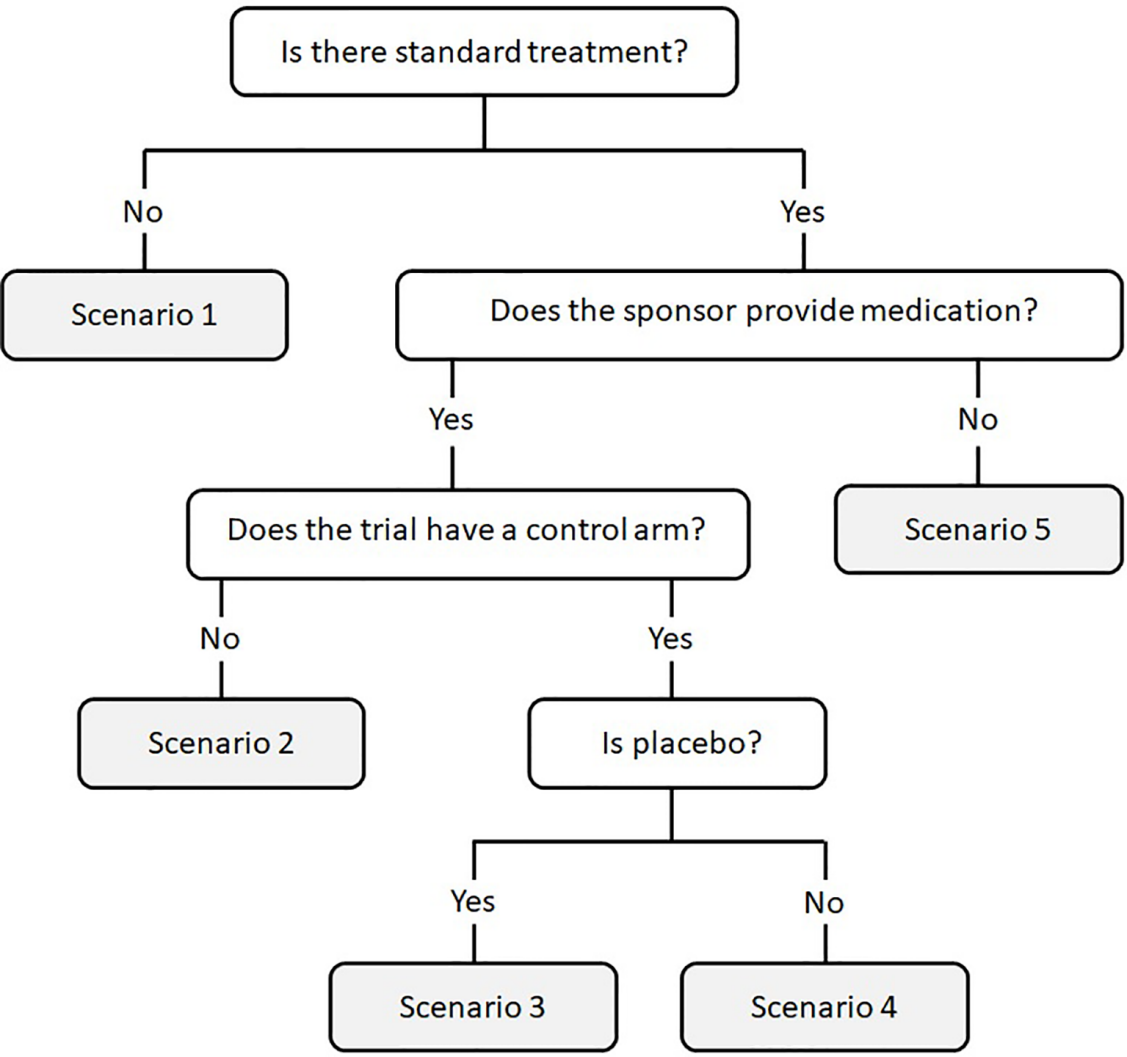
Decision tree algorithm to determine if a clinical trial results in drug cost avoidance. Scenarios 2, 3, and 4 establish that the clinical trial generates drug cost avoidance, while scenarios 1 and 5 generate no savings.

Following the previous definitions, a decision tree algorithm was created based on the availability or not of standard treatment, the presence or absence of a control group (placebo or active treatment), and the provider of the medication (private sponsor or hospital-acquired). This algorithm defines five scenarios to classify clinical trials and their potential savings (**Figure 1**): 1) There is no standard treatment; 2) There is standard treatment, the sponsor provides some or all of the medication, and there is no control group; 3) There is standard treatment, the sponsor provides some or all of the medication, and control group is placebo; 4) There is standard treatment, the sponsor provides some or all of the medication, and the control group is different from placebo; 5) Medication is not provided by the sponsor (e.g., academic trials without a sponsor). According to the previous definitions, scenarios 2, 3, and 4 were considered to generate drug cost avoidance, while scenarios 1 and 5 generate no savings.

### Variable Selection and Data Standardization

Once the scenario is assigned, our methodology is based on estimating the drug cost avoidance. In this step, we collected or calculated the following parameters for each clinical trial:

* Information about the investigational drug and the standard treatment: name of drugs, dosing regimen (including loading dose when needed), length of cycle, treatment duration (fixed duration or until tumor progression).
* Provider of the medication (private sponsor or hospital): sponsors could either provide the medications directly to the hospital or refund their use. In addition, they could either provide some or all of the medications.
* To determine the standard treatment, the National Comprehensive Cancer Network (NCCN) guidelines (20) and the Investigator’s Brochure information were consulted. Only one approved standard treatment was selected for each clinical trial. The only exception to this was for the trials with substantial differences between the standard treatments that could be administered to patients (for example, in trials targeting both squamous and non-squamous lung cancer patients). The selection of the standard treatment was revised by a panel of subject matter experts composed of oncologists and oncology pharmacists. We selected the most used treatment according to our hospital protocols and records when more than one standard of care could be considered. Multi-tumor clinical trials were excluded from the economic analysis due to the difficulty of selecting a unique standard treatment.
* Drug doses were calculated considering the following assumptions:
o A standard weight of 70 kg and a body surface area of 1.70 m^2^ were established.
o Dose reductions or treatment interruptions were not considered.
o Doses were calculated considering the dosing regimen stated in the Investigator’s Brochure or the summary of product characteristics.
* In terms of treatment duration, the median number of cycles or the median days on treatment were considered for the standard treatment, according to the literature. When this information was not available, and the duration of the treatment was determined by disease progression, the median progression-free survival was considered. Results from individual clinical trials were considered to establish the treatment duration.
* Costs were considered from a National Health System perspective, using notified sale prices, including taxes (4% VAT) and a 7.5%-15% rebate (according to the national Royal Decree-Law 8/2010). We excluded local negotiated discounts. We considered the cost established for each drug by 1 January. Costs are reported in euros.

### Cost Analysis

We created a spreadsheet with the equations needed to calculate the drug cost avoidance based on data collected for each clinical trial and the scenario described in the decision algorithm.

The drug cost avoidance was calculated as follows:

1. Calculation of the received dose.
2. Calculation of the cost of standard treatment and investigational drugs not provided by the sponsor.
* Cost per dose: Cost per milligram of drug * total dose received per administration. Drug wastage was not considered.
* Cost per cycle: Cost per dose * number of doses per cycle.
* Cost per patient: Cost per cycle * number of received cycles (alternative for oral treatments: cost per day * days of treatment). The whole number of cycles was used.

We considered drug cost avoidance per clinical trial as the difference between the drug cost of treating a patient with the local standard treatment and the drug cost for our hospital of treating the same patient if enrolled in the trial. An example of the calculations needed to determine the drug cost avoidance is detailed in **Table 1**.

**Table 1:** Example of calculation of drug cost avoidance per clinical trial.

Characteristic	
Clinical trial protocol’s code	BO29159
Arms Single-arm	Trastuzumab SC 600 mg day 1 + Pertuzumab 420 mg day 1 (loading dose: 840 mg) + Docetaxel according to clinical practice day 1. A cycle of 21 days.
Investigational treatment information	
Drugs provided by the sponsor	Trastuzumab SC and Pertuzumab (docetaxel is not provided)
Duration of treatment	Until tumor progression
Standard treatment information	
Best Standard of Care	Trastuzumab IV 6 mg/kg (loading dose: 8 mg/kg) + Pertuzumab 420 mg (loading dose: 840 mg) + Docetaxel 75 mg/m^2^. A cycle of 21 days.
Reference	NCCN guidelines (20)
Duration of treatment	Until tumor progression
No. of cycles according to bibliography	24
Reference	CLEOPATRA study (N Engl J Med 2015; 372: 724-734)
Scenario from the decision algorithm	2
Cost to the pharmacy department of a patient enrolled in the clinical trial	
Docetaxel	
Cost/mg Dose Cost/dose Cost/cycle Cost/patient	€1.1432 75 mg * 1.70 m^2^ = 127.5 mg 127.5 * €1.1432 = €145.76 €145.76 €145.76 * 24 = €3,498.19
Cost to the pharmacy department of a patient treated with the local standard treatment	
Trastuzumab IV	
Cost/mg Dose Cost/dose Cost/cycle Cost/patient	€2.8952 6 mg * 70 kg = 420 mg (loading dose: 560 mg) 420 * €2.8952 = €1,215.97 (loading dose: €1,621.29) €1,215.97 €1,621.29 + 23 * €1,215.97 = €29,588.55
Pertuzumab	
Cost/mg Dose Cost/dose Cost/cycle Cost/patient	€6.9333 420 mg (loading dose: 840 mg) 420 * €6.9333 = €2,911.98 (loading dose: €5,823.95) €2,911.98 €5,823.95 + 23 * €2,911.98 = €72,799.35
Docetaxel Cost/patient	€3,498.19
Total	€29,588.55 + €72,799.35 + €3,498.19 = €105,886.10
Drug cost avoidance	€105,886.10 – €3,498.19 = **€102,387.91**

IV, intravenous; SC, subcutaneous.

### Feasibility Study of the Methodology

We conducted a cross-sectional study in a tertiary hospital of the public health system, serving a population of more than 350,000 inhabitants in Madrid, Spain. We reviewed all oncology clinical trials opened to recruitment in 2019, and all active and recruiting oncology clinical trials from previous years with at least one patient in 2019.

Data were collected from the pharmacy software for investigational drug accountability and dispensing log (pkEnsayos^®^). In addition to the variables necessary for the cost analysis described above, we collected the following variables for each clinical trial:

* Tumor type: digestive tumors, genitourinary tumors, breast cancer, melanoma, lung cancer, multi-tumor, other.
* Protocol code, phase (I, I/II, II, II/III, III), and indication.

First, we estimated the drug cost avoidance per clinical trial, classified by type of tumor, phase of the study, and scenario from our decision algorithm. Average drug cost avoidance was reported as mean value and range. Then, we estimated the total drug cost avoidance in our hospital, taking into consideration the actual number of patients enrolled in each trial during 2019. For this, we multiplied the drug cost avoidance of each trial by the number of patients enrolled in that trial. Finally, we estimated the average drug cost avoidance per patient, classified by type of tumor, phase of the study, and scenario.

## Results

This methodology was implemented in our investigational drug service (IDS) in January 2019. We describe here the results derived from our feasibility study. A total of 159 active oncology clinical trials were identified. The main characteristics of these trials are summarized in **Table 2**. Genitourinary and digestive tumors were the most frequent tumor locations. Over half of the trials were phase III (88; 55.3%), and a little less than half (76; 47.8%) were activated in our center during 2019. Overall, 236 patients were enrolled in oncology clinical trials in 2019, representing around 15% of oncology patients receiving active treatment in our center.

**Table 2:** Characteristics of the analyzed clinical trials. Data are n (%).

Characteristic	All trials (*N* = 159)	Excluding multi-tumor trials (*N* = 140)
Type of tumor		
Genitourinary tumors	38 (23.9)	38 (27.1)
Digestive tumors	32 (20.1)	32 (22.9)
Breast cancer	26 (16.3)	26 (18.6)
Lung cancer	23 (14.5)	23 (16.4)
Multi-tumor	19 (11.9)	–
Melanoma	15 (9.4)	15 (10.7)
Other^a^	6 (3.8)	6 (4.3)
Phase of investigation		
I	21 (13.2)	9 (6.4)
I/II	14 (8.8)	10 (7.1)
II	32 (20.1)	29 (20.7)
II/III	4 (2.5)	4 (2.9)
III	88 (55.3)	88 (62.9)
Scenario from the decision algorithm^b^		
1		15 (10.7)
2		33 (23.6)
3		1 (0.7)
4		86 (61.4)
5		5 (3.6)

a Other group includes sarcoma (3), head and neck cancer (1), glioblastoma (1) and Merkel-cell carcinoma (1).

b The decision algorithm was not applied to multi-tumor trials.

We excluded for further analysis multi-tumor trials (19; 11.9%). More than a half of these trials (10/19; 52.6%) stated in their inclusion criteria that they are for patients not candidates for standard treatment. We applied our decision algorithm to the 140 remaining clinical trials. Results are shown in **Table 2**. There was no drug cost avoidance in 20 trials (14.3%): no standard treatment (scenario 1) in 15 trials (10.7%), and no provision of drugs by the sponsor (scenario 5) in 5 trials (3.6%). Drug cost avoidance was found in the remaining 120 trials (85.7%). From these trials aligning with scenarios 2-4, the sponsor provided all of the medication in 111 (92.5%) and part of the medication in 9 (7.5%).

**Figure 2:**
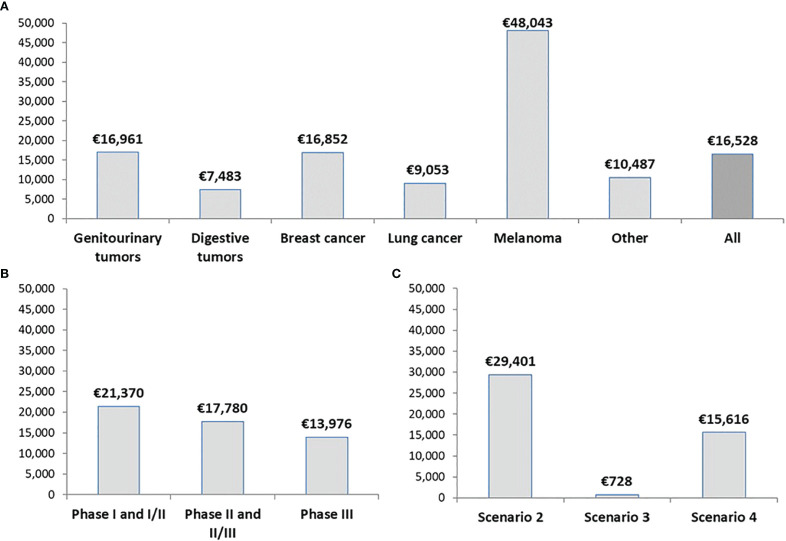
Average drug cost avoidance per oncology clinical trial. Data are classified by **(A)** Type of tumor, **(B)** Phase of the study, and **(C)** Scenario from our decision algorithm. The actual number of patients enrolled in each trial is not considered.

**Figure 2** shows the average drug cost avoidance per clinical trial. We define it as the estimated drug cost avoidance when one patient is enrolled in that trial. Overall, we estimated an average drug cost avoidance per trial of €16,528 (range: 0 – €105,227). Analyzing these data per type of tumor (**Figure 2A**), we observed that melanoma clinical trials are the ones that produced the highest average cost avoidance (mean: €48,043; range: 0 – €105,227), followed by genitourinary tumors (mean: €16,961; range: 0 – €64,434), breast cancer (mean: €16,852; range: 0 – €102,388), lung cancer (mean: €9,053; range: 0 – €35,222) and digestive tumors (mean: €7,483; range: 0 – €33,600). We also classified the drug cost avoidance per trial according to the study phase (**Figure 2B**). Early-phase trials (I and I/II; n=19) were the studies with the highest average drug cost avoidance (mean: €21,370; range: 0 - €85,615), followed by phases II and II/III trials (n= 33; mean: €17,780; range: 0-85,615€), and phase III trials (n=88; mean: €15,185; range: 0 - €105,227). Finally, according to the scenario from our decision algorithm, it was observed a higher average drug cost avoidance in scenario 2 studies (mean: €29,401; range: €171 - €102,388) than in scenario 4 studies (mean: €15,616; range: €270 - €105,227) (**Figure 2C**).

**Figure 3:**
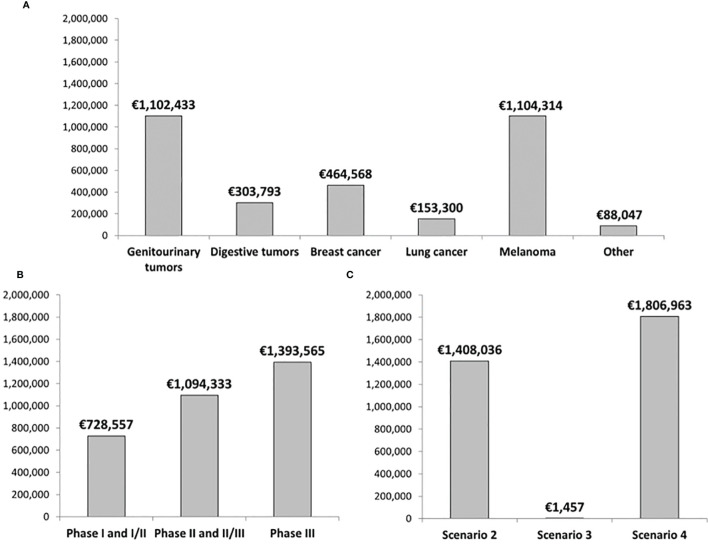
Total drug cost avoidance classified by **(A)** Type of tumor, **(B)** Phase of the study, and **(C)** Scenario from our decision algorithm. Data corresponds to the total number of patients enrolled in oncology clinical trials during 2019 in our hospital.

Overall, 198 patients were enrolled during 2019 in the 140 oncology clinical trials analyzed in our study. We estimated that the total drug cost avoidance in our hospital amounts to €3,216,456, considering the trial in which each patient was enrolled. **Figure 3A** shows these savings classified by type of tumor. Melanoma (n=27 patients) and genitourinary tumors (n=61) were the tumor types associated with the highest drug cost avoidance (more than €1,100,000). Most patients (n=117; 59.1%) were enrolled into Phase III trials. As observed in **Figure 3B**, these studies produced a drug cost avoidance of nearly €1,400,000. Finally, **Figure 3C** shows the drug cost avoidance of trials aligning with scenarios 2-4. Two patients were enrolled in the only study aligning with scenario 3.

**Figure 4:**
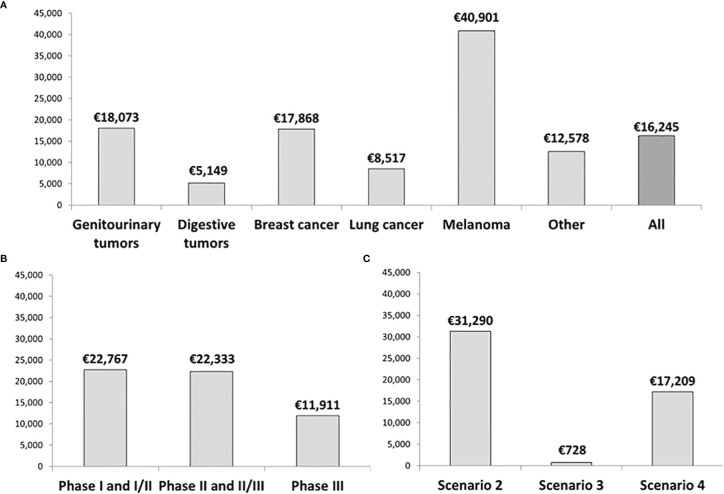
Average drug cost avoidance per patient classified by **(A)** Type of tumor, **(B)** Phase of the study, and **(C)** Scenario from our decision algorithm. Data corresponds to the total number of patients enrolled in clinical trials during 2019 in our hospital.

The average drug cost avoidance per patient enrolled in a clinical trial was €16,245 (n=198 patients). **Figure 4A** shows that the tumor types with the highest drug cost avoidance per patient are melanoma (€40,901 per patient), genitourinary tumors (€18,073 per patient) and breast cancer (€17,868 per patient). Average drug cost avoidance per patient was significantly higher in early-phase trials than Phase III trials (**Figure 4B**). Finally, trials aligning with scenario 2 produced the highest average drug cost avoidance per patient (**Figure 4C**).

## Discussion

The provision of drugs free of charge by pharmaceutical companies has been identified as the primary source of cost-savings in clinical trials (13). The growing number of clinical trials and their increasing complexity encourages the development of a methodology that can be used systematically to estimate drug cost avoidance. Our algorithm allows determining whether a clinical trial will produce any drug cost saving or not. We believe that considering as a reference the cost of the standard treatment that the patient would have received if had not been enrolled in the trial (with the most usual doses and duration) can show the real savings that a clinical trial can produce. Some studies have estimated the drug cost avoidance based on the price of investigational drugs, and when this price is not available, taking as a reference the price of a control drug or standard treatment (11, 18, 21). However, some other studies like ours have used the standard therapy as a comparator (10, 12, 16), which we believe provides a more comprehensive analysis since clinical trials with novel agents with price not available are not excluded, as observed in other studies (11, 14, 15). In fact, among all the active oncology clinical trials in our hospital, only the multi-tumor trials were excluded from the economic analysis since a suitable comparator was not found. Patients enrolled in these trials have very different tumor types and in many cases are not candidates for standard treatment, so it is difficult to define a single standard therapy.

We standardized some of the variables (for example: body surface area, weight, or median of cycles received) instead of analyzing them individually (10, 12, 15, 17). In this way, the drug cost avoidance produced by the clinical trial can be estimated even before the recruitment of patients. We propose using the median number of cycles, or the median days on treatment according to the existing literature for each tumor and setting to determine the duration of the standard treatment. We consider this an essential difference from previous studies. For example, Bredin et al. (10) assessed the actual drug cost avoidance considering the standard-of-care dosing regimen over the period the patient was administered the investigational drug. In addition, they also estimated the potential drug cost avoidance, considered as the cost of treatment if the patient had remained on trial for the protocol specified study length.

To our knowledge, this is the most extensive study on drug cost avoidance resulting from oncology clinical trials in terms of the number of clinical trials. From the 159 oncology clinical trials active in our center during 2019, we report that 120 (75.5%) resulted in drug cost avoidance. This is a significantly higher percentage compared to previously published studies. Bredin et al. reviewed 101 studies conducted during 1992-2007, noting that 42 (41.6%) provided drug cost avoidance (10). Tang et al. (15) reported that only 17 (14.5%) out of 117 clinical trials conducted from 1999 to 2011 resulted in drug cost avoidance. Other recent studies have included a small number of clinical trials focusing on a specific tumor type (12, 14). We think that our methodology could be useful for conducting multicenter studies to analyze the causes of these notable differences. It could be applied by any IDS to determine the consistency of our results and to know more accurately the economic impact of clinical trials.

The avoided costs estimated in this work fully benefit our hospital and our health system. When evaluating the cost of clinical trials for institutions, defining the economic benefit of drug cost avoidance seems to be essential. Drug cost avoidance could offset the expenditures required to conduct clinical trials. IDS should participate in scientific review committees to evaluate the priority and feasibility of research protocols (22, 23). Standardizing these processes could improve efficiency, so our methodology could have a particular value when conducting an assessment prior to supporting the protocol. This study shows that oncology clinical trials in which drugs are provided free of charge or refunded by the sponsor imply substantial economic savings for hospitals. In our hospital, the pharmaceutical expenditures in cancer treatments in 2019 amount to nearly €20 million. Since this opportunity to reduce drug costs has been identified, we believe it is indispensable to dedicate sufficient resources to promote clinical trials.

Among the limitations of our methodology, we must emphasize that drug cost avoidance can only be an estimate. An assessment of the accuracy and reliability of the assumptions made in this methodology has not yet been performed. We believe that conducting multicenter studies would be the better strategy to carry out these assessments. The duration of cancer treatment can be very variable for each patient, especially in the palliative setting, and the calculation of the avoided cost could be overestimated because no dose reductions or treatment interruptions were taken into account. To minimize these limitations, we considered the median number of cycles received in pivotal trials (when possible) for the comparator. Another limitation is the availability of more than one standard therapy for certain indications, so the choice could be influenced by each center’s usual practice and clinicians. To reduce the potential for selection bias, a panel of subject matter experts composed of oncologists and oncology pharmacists reviewed the selection of the standard treatment. Finally, cost savings in our study are exclusively estimated as drug cost avoidance; we did not consider other costs related to the screening and conduction of clinical trials, or other sources of costs such as those associated with patient care: visits to the outpatient clinic for therapy or appointments, drug administration costs, treatment of adverse events, or supportive treatments.

## Conclusions

In conclusion, we describe a standardized method to estimate drug cost avoidance in clinical trials. Although this work has been applied to oncology clinical trials, its design allows it to be used in any disease. We have estimated that our hospital’s total drug cost avoidance associated with all patients enrolled during 2019 in an oncology clinical trial amount to €3,216,456 (€16,245 per patient). This methodology would allow making comparisons between different healthcare providers.

## Data Availability Statement

The raw data supporting the conclusions of this article will be made available by the authors, without undue reservation.

## Author Contributions

SGS: Investigation, Formal analysis, Writing original draft. RC-B: Conceptualization, Methodology, Writing – Review and Editing. EG-H: Writing – Review and Editing. JLR-H: Writing – Review and Editing. All authors helped design the methodology and research, critically revised the manuscript, and approved the final version to be published.

## Conflict of Interest

The authors declare that the research was conducted in the absence of any commercial or financial relationships that could be construed as a potential conflict of interest.

## Publisher’s Note

All claims expressed in this article are solely those of the authors and do not necessarily represent those of their affiliated organizations, or those of the publisher, the editors and the reviewers. Any product that may be evaluated in this article, or claim that may be made by its manufacturer, is not guaranteed or endorsed by the publisher.

## Abbreviations

IDS, Investigational drug service; NCCN, National Comprehensive Cancer Network.
